# An Unusual Case of Tertiary Syphilis Behaving Like Tongue Squamous Cell Carcinoma

**DOI:** 10.1177/2324709618820355

**Published:** 2018-12-20

**Authors:** Roberto N. Solis, Brooks T. Kuhn, D. Gregory Farwell

**Affiliations:** 1 Texas Tech University Health Sciences Center, El Paso, TX, USA; 2 Division of Pulmonary Medicine, Department of Internal Medicine, University of California, Davis, Sacramento, CA, USA; 3 Department of Otolaryngology-Head and Neck Surgery, University of California, Davis, Sacramento, CA, USA

**Keywords:** syphilis, tertiary syphilis, tongue, oral manifestations, granulomatous inflammation

## Abstract

Syphilis may present with a myriad of oral manifestations in the primary, secondary, and tertiary stages, and may be confused with malignancy. Despite a rise in the incidence of syphilis, tertiary syphilis is exceedingly rare. Tertiary syphilis gummas usually affect the hard palate, while tongue involvement is very rare. A 55-year-old male with extensive smoking and alcohol use was referred for malignancy evaluation with an ulcerative mass creating a tongue cleft, and a positron emission tomography scan suggestive for malignancy. Biopsy results demonstrated no carcinoma but histology demonstrated granulomatous inflammation. Further laboratory results demonstrated elevated rapid plasma reagin titers with *Treponema pallidum* immunoglobulin G antibodies present. The patient was diagnosed with tertiary syphilis, received appropriate antibiotic therapy, and had healing of the tongue with a persistent cleft. Syphilis may mimic many disease processes. As such, it is important to include this disease in the differential of an unusual tongue lesion. An oral lesion may be the first sign of infection.

## Introduction

Syphilis is caused by *Treponema pallidum*, an anaerobic spirochete, and has long been referenced as the “great imitator” for affecting a number of organs with variable presentations.^1^ With the rise in incidence of reported cases of syphilis in the United States, it is important to be aware of the oral manifestations of this disease.^2^ Oral lesions of syphilis manifest differently in the primary, secondary, and tertiary stages, and may be the first sign of syphilis.^3^ We present the case of a 55-year-old man with a history of heavy smoking and alcohol who presented with a painful, deep ulcerative lesion of the left anterior tongue, very suspicious for squamous cell carcinoma. Multiple biopsies confirmed no evidence of tumor, and additional testing demonstrated the ulcerative lesion was an oral manifestation of tertiary syphilis.

## Case Report

A 55-year-old man with a history of extensive alcohol and tobacco use presented with a 2-month history of a progressively enlarging, 5-cm ulcerative, and painful midline tongue lesion extending to the floor of mouth resulting in an anterior tongue cleft. This lesion was associated with unintentional weight loss, left otalgia, and submandibular swelling. Bilateral nontender palpable lymphadenopathy in levels I, II, and III were present.

**Figure 1.**
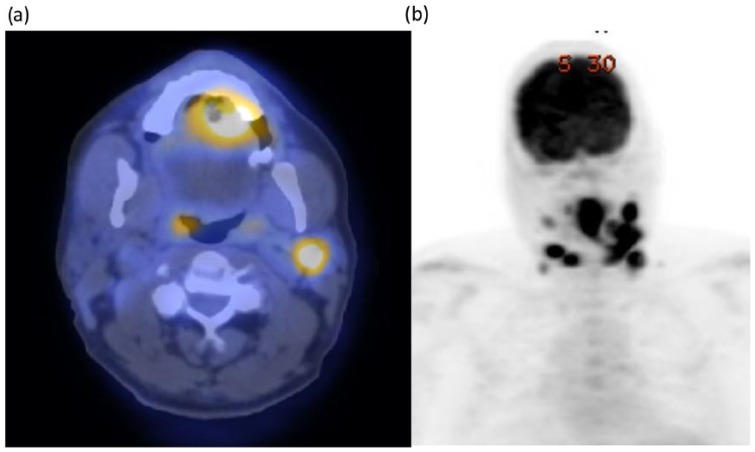
(a) Positron emission tomography (PET) scan demonstrates hypermetabolic lesion in the tongue, which was suspicious for malignancy. (b) PET scan demonstrates multiple cervical lymph node involvement, which was suspicious for malignancy.

Prior biopsies by an otolaryngologist in private practice did not demonstrate carcinoma but were otherwise inconclusive with reactive inflammatory changes. However, a positron emission tomography scan revealed hypermetabolic lesions of the anterior tongue as well as lymph nodes bilaterally in levels I, II, and III suggestive of malignancy (Figure 1). The assessment was a likely T3/4N2cM0 tongue squamous cell carcinoma. He underwent a panendoscopy with biopsies and a physical examination under anesthesia for surgical planning. Biopsy results again did not yield carcinoma but showed granulomatous inflammation without organisms present.

**Figure 2.**
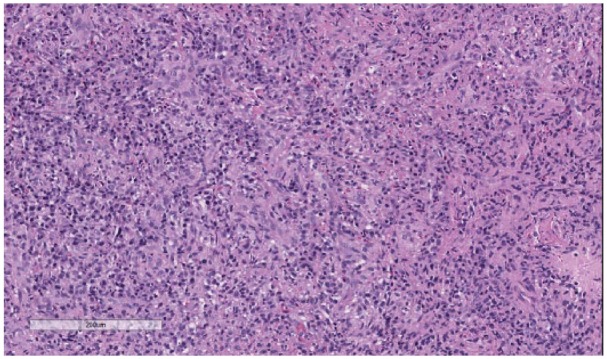
Lymph node tissue sample with hematoxylin-eosin stain demonstrating granulomatous inflammation.

**Figure 3.**
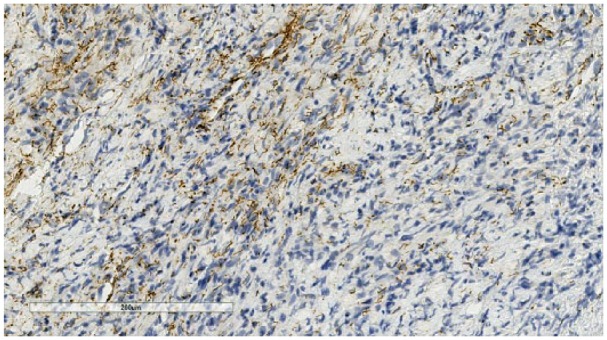
Lymph node tissue sample with immunohistochemistry demonstrating spirochetes.

**Figure 4.**
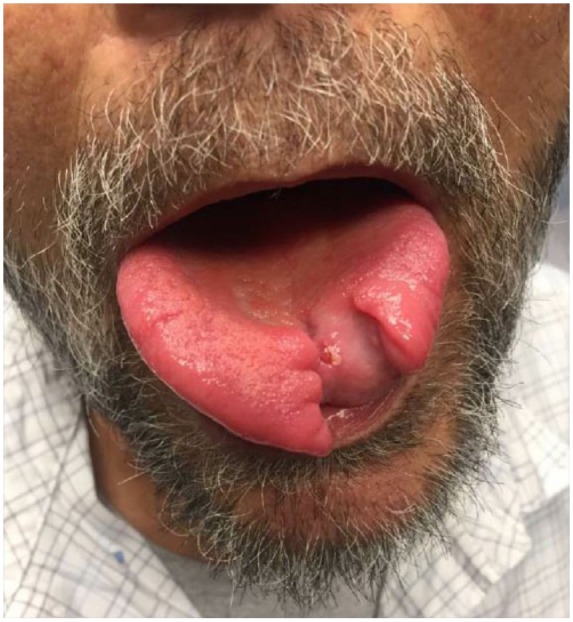
Persistent tongue cleft after antibiotic therapy.

A multidisciplinary tumor board recommended a rheumatologic workup and an excisional lymph node biopsy. Lymph node biopsy results demonstrated noncaseating granulomatous inflammation with no malignancy (Figure 2), while laboratory results demonstrated markedly elevated ACE (angiotensin converting enzyme) levels. These findings suggested an atypical case of sarcoidosis, and the patient was referred for pulmonary consultation. The patient had developed scattered erythematous macules involving the extremities, palms, soles, and trunk. Laboratory evaluation demonstrated *T pallidum* immunoglobulin G antibodies present with a reflex RPR (rapid plasma reagin) titer of 1:512. The lymph node biopsy sample was then analyzed with immunohistochemistry (IHC) revealing spirochetes (Figure 3). The patient was diagnosed with tertiary syphilis and started on doxycycline 100 mg twice daily for 30 days because of a severe penicillin allergy. After receiving treatment, the patient came back to our clinic with his tongue lesion healing well but with a persistent anterior tongue cleft (Figure 4). The patient has not obliged with further laboratory evaluations to recheck a RPR titer after antibiotic therapy.

## Discussion

Despite the rise in incidence of syphilis, tertiary syphilis is exceedingly rare. Successful syphilis-control programs and the widespread use of antibiotics for other conditions that will indirectly eradicate latent syphilis have led to this improvement. While primary syphilis is characterized by primary skin lesions at the site of inoculation around 21 days after exposure, secondary syphilis has a wide spectrum of manifestations because of hematogenous spread.^1^ The oral manifestations of secondary syphilis are common and have been well described in the literature. They include multiple, often symptomatic elevated plaques with occasional ulceration with a pseudomembrane.^4-6^ Syphilis will progress to the tertiary stage in one third of untreated patients after several years of latency.^1^ Tertiary syphilis is defined by painless gummas, which are granulomatous-like lesions that can involve any organ and can range from small, superficial lesions to large ulcerative masses. Additionally, tertiary syphilis can present with cardiovascular and neurological manifestations.^7^

In a case series and review of the oral manifestations of syphilis by Leuci et al, 6 cases of tertiary syphilis involving the oral cavity were described. Four involved the hard palate, 1 created a cleft in the soft palate, and in 1 case they describe a necrotic tongue lesion affecting the dorsum of the tongue.^6^ In a case series from 1950 to 1965 performed by Meyer and Shklar,^3^ 68 cases of tertiary syphilis involving the oral cavity are described ranging from small, raised lesions to large lesions with tissue necrosis leading to bony destruction. This case series notes the hard palate as the most frequently affected site, while atrophic glossitis was the most characteristic lesion in the oral cavity. The authors also mention that in many cases carcinoma was presumed but biopsy results resulted in granulomatous inflammation. Of note, there were no ulcerative lesions involving the tongue in this case series as seen in our patient.^3^

A biopsy may be the first diagnostic approach in patients showing an atypical presentation of syphilis as in the case of our patient. Usually histopathologic findings may be nonspecific, especially in the primary and secondary stages of the disease; however, the tertiary stage has been noted to demonstrate granulomatous inflammation with or without necrosis.^8^ While direct visualization of spirochetes is possible in the primary and early secondary stages of syphilis, *T pallidum* is rarely visualized in the tertiary stage, even with the use of special stains.^3,7,8^ IHC detects spirochetes in tissue samples with a sensitivity of 71% to 94% in primary syphilis and early secondary syphilis^9^; however, in later stages, IHC loses sensitivity because of fewer organisms in tissues.^10^ We were able to identify spirochetes in our lymph node tissue sample, despite the lower sensitivity seen in tertiary syphilis.

Granulomatous inflammation in the mouth is a rare occurrence with a wide differential including infectious and noninfectious etiologies (Table 1).^11,12^ Foreign bodies are the most common cause of granulomatous inflammation in the oral cavity, while tertiary syphilis is very rare.^11^

**Table 1.** The Most Common Type of Granulomatous Inflammatory Conditions in the Oral Cavity by Noninfectious and Infectious Etiologies.^11^

Noninfectious
Foreign body reaction
Sarcoidosis
Crohn disease
Orofacial granulomatosis
Infectious
Tuberculosis
Hansen’s disease
Tertiary syphilis
*Bartonella henselae*
Histoplasmosis
Cryptococcosis
Blastomycosis
Paracoccidioidomycosis

Our patient had a significant history of alcohol and tobacco abuse, a painful ulcerative tongue lesion, lymphadenopathy, and a positive positron emission tomography scan, which made us consider malignancy as our top differential even after 2 negative biopsies for malignancy. To our knowledge, this is the first case to be reported in which tertiary syphilis has created a tongue cleft in a patient. It is vital to have a wide differential in the setting of granulomatous inflammation seen on histopathology. Because syphilis presents in a myriad of ways, this disease poses a diagnostic challenge and will oftentimes take an extensive workup to lead to the diagnosis. Therefore, it is critical for clinicians to consider syphilis in the differential for ulcerative oral lesions and to perform serological tests in questionable cases.
